# Biallelic loss of function variants in *FUZ* result in an orofaciodigital syndrome

**DOI:** 10.1038/s41431-024-01619-6

**Published:** 2024-05-03

**Authors:** Swati Singh, Sheela Nampoothiri, Dhanya Lakshmi Narayanan, Chakshu Chaudhry, Sandesh Salvankar, Katta M. Girisha

**Affiliations:** 1 Department of Medical Genetics, Kasturba Medical College, Manipal, Manipal Academy of Higher Education, Manipal, Karnataka India; 2 Department of Paediatric Genetics, Amrita Institute of Medical Sciences and Research Centre, Kochi, India; 3 Suma Genomics Private Limited, Manipal, India; 4 Department of Genetics, College of Medicine and Health Sciences, Sultan Qaboos University, Muscat, Sultanate of Oman

**Keywords:** Pathogenesis, Genetics

## Abstract

Orofaciodigital syndrome is a distinctive subtype of skeletal ciliopathies. Disease-causing variants in the genes encoding the CPLANE complex result in a wide variety of skeletal dysplasia with disturbed ciliary functions. The phenotypic spectrum includes orofaciodigital syndrome and short rib polydactyly syndrome. FUZ, as a part of the CPLANE complex, is involved in intraflagellar vesicular trafficking within primary cilia. Previously, the variants, c.98_111+9del and c.851G>T in *FUZ* were identified in two individuals with a skeletal ciliopathy, manifesting digital anomalies (polydactyly, syndactyly), orofacial cleft, short ribs and cardiac defects. Here, we present two novel variants, c.601G>A and c.625_636del in biallelic state, in two additional subjects exhibiting phenotypic overlap with the previously reported cases. Our findings underscore the association between biallelic loss of function variants in *FUZ* and skeletal ciliopathy akin to orofaciodigital syndrome.

## Introduction

Skeletal ciliopathies are a diverse group of rare disorders [1]. These conditions are caused by either a defect in the formation of cilia or a dysfunction of centrosome or cilia. They are recognized by a broad range of phenotypes: short ribs, short limbs, short digits, polydactyly, trident ilia, cone shaped epiphyses and cupped metaphases. They also exhibit pleiotropy and affect the craniofacial, nervous, cardiac, gastrointestinal, and genitourinary systems [2–5].

The primary cilia is a non-motile protrusion from the cell surface of most mammalian cells. It is involved in cell signaling and development. The assembly of cilia requires synchronized involvement of several regulatory complexes and signaling proteins [6]. One of the essential yet least understood complexes is CPLANE (ciliogenesis and planar polarity effector). The core CPLANE complex consists of INTU (Inturned Planar Cell Polarity Protein), FUZ (Fuzzy Planar Cell Polarity Protein) and WDPCP (WD Repeat Containing Planar Cell Polarity Effector), which also strongly interacts with CPLANE1 and RSG1 (CPLANE2) [7]. Perturbation of the expression of these genes might cause disruption of ciliogenesis and result in skeletal ciliopathies (Short rib‐polydactyly syndrome (SRPS), INTU‐related (MIM# 617925), Congenital heart defects, hamartomas of tongue, and polysyndactyly, WDPCP-related (MIM# 217085), Joubert syndrome 17, CPLANE1-related (MIM# 614615) and Orofaciodigital syndrome VI, CPLANE1-related (MIM# 277170). The phenotypic spectrum is wide and includes short rib thoracic dysplasia and orofaciodigital syndrome.

Recently, *FUZ* has been implicated in vesicular trafficking within primary cilia [7, 8]. FUZ plays a critical role in transporting protein necessary for cilium assembly and cell signaling such as Wnt, FGF, hedgehog signaling pathways. The intricate network of multiple signaling pathways and interactions among germ layers and neural crest cells is crucial during the process of organogenesis and other embryonic developmental processes. Dysregulation of these networks due to disruption in ciliary function can contribute to various diseases. It has been observed that defects in *FUZ* are associated with severe neural tube defects, craniosynostosis, cardiac abnormalities, or skeletal dysplasia [8–12]. Only two patients have been described until now where biallelic loss of function variants in *FUZ* were expected to result in a skeletal ciliopathy [4]. Herein, we describe two Indian patients carrying biallelic variants in *FUZ* with digital anomalies, orofacial cleft, short ribs and cardiac defects resembling orofaciodigital syndrome.

## Methods

**Fig. 1:**
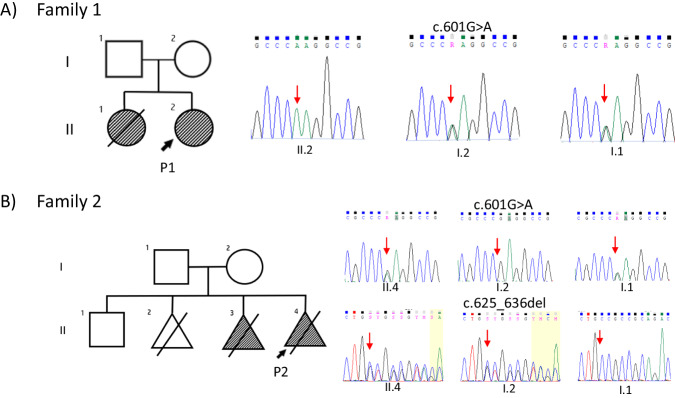
Pedigrees of two families with disease-causing variants in FUZ. Sanger sequencing chromatograms show segregation of these variants in both families.

**Fig. 2:**
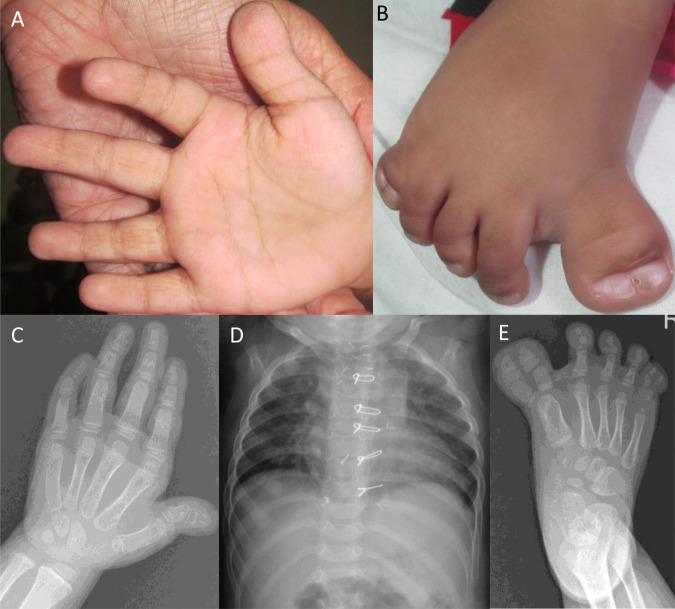
Clinical features of proband 1 at age 2 years and 9 months. Short and broad thumbs and clinodactyly of 5th finger were noted (**A** and **C**). Preaxial and postaxial polysyndactyly were observed in the right foot (**B** and **E**). Chest radiograph is unremarkable (**D**).

Two families were ascertained for clinical testing (Figs. 1 and 2) in view of skeletal dysplasia. Genomic DNA was isolated from the blood using QIAmp DNA Blood Mini Kit (Cat# 51106, QIAGEN, USA). Exome sequencing was performed using the protocol described previously [13]. Furthermore, data was annotated by ‘Annotate Variation (ANNOVAR)’ [14] and our in-house scripts [15]. The filtered variants were then analyzed using relevant genetic databases and coding/noncoding variant pathogenicity predictors. Sanger sequencing was carried out for the validation and segregation of the rare disease-causing variants identified in the families (Fig. 1). Homozygous regions for a minimum size of 1 Mb from the exome sequencing data of the probands were identified using AutoMap [16]. The variants were described according to HGVS nomenclature, using reference sequences NM_025129.5 (GRCh38) and NP_079405.2. Both the variants are submitted to ClinVar (SUB13866645 and SUB13866677).

## Results

### Family 1

The proband 1 is a two-years-nine-months old girl. Her birth weight was 2.8 kg (−1.19 SD). A congenital heart defect was noted during the antenatal scan. Her development and speech were normal. Her height was 94 cm (+0.37 SD), weight 10.5 kg (−2.24 SD) and head circumference 46 cm (−1.51 SD) at the time of examination. She had a prominent forehead, medial flaring of the eyebrow, low set ears with prominent antihelix, broad nasal bridge and narrow chest. Short and broad thumbs and clinodactyly of 5^th^ fingers were noted. Additionally, bilateral preaxial polysyndactyly was evident in feet and postaxial polysyndactyly in the right foot. She had a partial atrioventricular canal defect (AVCD) that was surgically corrected. The clinical and radiological findings of proband 1 are described in Fig. 2.

Her elder sister died at the age of 1.5 years and was found to be similarly affected. A cardiac evaluation had revealed a complete AVCD in her. Parents informed that she had bilateral cleft lip and palate, clinodactyly of the right 5th fingers and medially deviated, broad great toes. Her DNA was not available for testing.

Exome sequencing revealed a novel homozygous missense variant (c.601G>A, p.(Glu201Lys) in exon 6 of *FUZ*. Sanger sequencing confirmed the presence of this variant in heterozygous state in her parents (Fig. 1). This variant is observed in four individuals in the gnomAD database (v2.1.1) in heterozygous state with an allele frequency of 0.0000131. MutationTaster, SIFT, and Polyphen predicted this variant to be disease causing and it has a CADD phred score of 25, suggesting it to be deleterious. The multiple sequence alignment tool predicted wild type amino acid to be conserved in several species (Supplementary Fig. 1).

### Family 2

The parents of proband 2 were clinically unaffected and had: a five-year-old healthy son, a second trimester miscarriage at 16 weeks (no diagnosis), third pregnancy terminated at 19 weeks due to atrioventricular septal defect (AVSD), single umbilical artery and presence of polydactyly. In the fourth conception, at 25 years of the mother’s age, the antenatal scan showed a right cleft lip, polydactyly (seven digits in both hands and feet), bilateral duplicated hallux and AVSD in proband 2 at 19 weeks of gestation. The pregnancy was medically terminated, and the genomic DNA was tested.

Compound heterozygous variants were identified in exon 6 of *FUZ*: a in-frame deletion variant inherited from the mother c.625_636del, p.(Val209_Leu212del) and a missense variant c.601G>A, p.(Glu201Lys) inherited from the father (Fig. 1). Segregation analysis could not be performed in the healthy sibling as his DNA sample was unavailable for testing. The variant c.601G>A was also found in the unrelated family 1. The variant c.625_636del was absent in gnomAD database (v2.1.1) and results in in-frame deletion leading to change in protein length. The clinical and molecular findings of both probands and those reported earlier are summarized in Table 1. The variant c.601G>A resides in an autozygous region region in family 1 and shares the same haplotype with family 2 indicating a shared ancestry (Supplementary Tables 1 and 2).

**Table 1:** Summary of clinical and genetic findings of the patients affected with FUZ-related autosomal recessive disorder.

Authors	Present study		Zhang et al. [4]	
**Patient Id**	P1	P2	R11-569	R98-523A
**General characteristics**				
Age	2 years 9 months	19 weeks of gestational age	24 weeks of gestational age	NA (alive)
Gender	Female	ND	NA	NA
Ethnicity	Indian	Indian	African American	Caucasian
Consanguinity	+	−	−	NA
**Growth parameters**				
Weight (SD)	10.5 kg (−2.24)	ND	NA	NA
Height (SD)	94 cm ( + 0.37)	ND	NA	NA
Head circumference (SD)	46 cm (−1.51)	ND	NA	NA
**Craniofacial features**				
Midline facial cleft	−	ND	+	NA
Prominent forehead	+	ND	−	NA
Medial flaring of the eyebrow	+	ND	−	NA
Low set ears	+	ND	−	NA
Prominent antihelix	+	ND	−	NA
Cleft lip	−	+	−	NA
Broad nasal bridge	+	ND	−	NA
**Skeletal findings**				
Short ribs	−	ND	+	NA
Short and broad thumbs	+	ND	−	NA
Clinodactyly of 5th fingers	+	ND	−	NA
Polydactyly	+	−	+	+
Preaxial polysyndactyly of the foot	+	+	−	NA
Postaxial polysyndactyly of the foot	+	−	−	NA
**Cardiac anomalies/other findings**				
Thickened nuchal fold	−	−	+	NA
Atrioventricular canal/ septal defect	+	+	+	NA
Hypoplastic left ventricle	−	−	+	NA
Hypoplastic kidneys	ND	ND	+	NA
**Molecular findings in FUZ**				
Nucleotide change (NM_025129.5)	c.601G>A	c.625_636del, c.601G>A	c.98_111+9del	c.851G>T
Zygosity	Homozygous	Compound heterozygous	Homozygous	Heterozygous
Amino acid change (NP_079405.2)	p.(Glu201Lys)	p.(Val209_Leu212del), p.(Glu201Lys)	−	p.(Arg284Leu)
Exon	6	6	1	6
Domain	2	2	1	2

+ present, − absent, *ND* Not determined, *NA* Not available.

## Discussion

In this study, we describe two Indian families with phenotypes suggestive of a skeletal ciliopathy implicated by biallelic variants in *FUZ*. Both the families had multiple affected fetuses individuals, though genetic testing was performed only in the probands and parents.

*FUZ* has recently been associated with skeletal ciliopathy in humans [4, 8]. Digital anomalies (polydactyly, syndactyly), orofacial cleft, short ribs and cardiac defects are the major clinical findings in the patients (including this report) which has resemblance to the genetically heterogeneous orofaciodigital syndromes (Table 1). Four variants (two missense and two predicted loss of function), in biallelic state in *FUZ* in these patients suggest loss of function of FUZ as the likely cause of the skeletal phenotype in these families (Table 1 and Supplementary Fig. 2).

Possible role for FUZ in skeletal involvement was discovered earlier when *Fuz* knockout mice showed severe developmental abnormalities comprising craniofacial defects, malformed sternum, ribs and long bones, polydactyly, and incompletely penetrant rostral neural tube closure defects [10, 11, 17]. Currently known functions of CPLANE complex and the phenotypes that result from deficiency of the component proteins (listed above in the introduction) predict a skeletal ciliopathy for the deficiency of FUZ too, consistent with our observations.

Zhang et al., described a bi-allelic splice variant c.98_111+9del leading to frameshift deletion and likely loss of function of FUZ. This variant led to the prenatal death of the patient. The phenotypes of the patient comprised a long, narrow chest, moderately short ribs, short long bones, and extreme polydactyly of all four limbs, ventricular septal defect, hypoplastic kidneys, midline facial cleft, thickened nuchal fold and dilated third ventricle [4].

Similarly, in-frame deletion variant c.625_636del, detected in compound heterozygous state in family 2 is predicted to cause in-frame deletion. The impact of the variant, whether it leads to a less functional protein with an internal alteration or results in instability and loss of the gene product, remains unknown. The predicted consequence of in-frame deletion is retention of an altered protein product.

The missense variant c.851G>T, p.(Arg284Leu) was also reported by Zhang et al. in 2018 in a patient who had features of asphyxiating thoracic dystrophy/short-rib thoracic dysplasia with polydactyly, though they did not identify a second variant [4]. Another biallelic variant, p.(Arg284pro) with a different amino acid change at the same position was noted by Barrel et al. in 2021 in a pair of twins who had craniosynostosis and dilatation of the lateral brain ventricles with no other skeletal manifestations [8]. Functional assays demonstrated that this variant could partially rescue Fuz mutant ciliogenesis but showed impaired hedgehog signaling transduction. They further established that Fuz mutant osteoblasts had increased potential of mineralization compared to the wildtype which might affect craniofacial ossification. This variant was located in domain 2 of FUZ, which is upstream of C-terminal domain. C-terminal domain is predominantly involved in vesicular trafficking, critical for ciliogenesis [17, 18]. Association of heterozygous variants in *FUZ* with neural tube defect observed by Seo et al. has not been substantiated yet by other studies [12].

Both the missense p.(Glu201Lys) and in-frame deletion p.(Val209_Leu212del), may have a less deleterious effect than a null variant c.98_111+9del. which might explain the milder skeletal phenotype of orofaciodigital syndrome observed in surviving probands in the study. This is along the expected lines in the spectrum of skeletal ciliopathies where boundaries between the several phenotypic descriptions are indistinct. For instance, biallelic variants in *CEP120* results in Joubert syndrome 31 and severe short-rib thoracic dysplasia type 13 while spectrum of *KIAA0753* related conditions extends to Joubert syndrome 38, orofaciodigital syndrome XV andshort-rib thoracic dysplasia 21without polydactyly [1, 19, 20]. Further clinical and mechanistic studies would help in understanding the genetic defects and their relationship to the phenotype. In conclusion, this study provides clinical and genetic evidence for autosomal recessive *FUZ*-related skeletal ciliopathy.

### Web resources

PRIMER 3v.4.1.0, http://primer3.ut.ee/

Ensembl, https://asia.ensembl.org/index.html

NCBI, https://www.ncbi.nlm.nih.gov/

Mutation Taster, http://www.mutationtaster.org/

OMIM, https://www.omim.org/

gnomAD, https://gnomad.broadinstitute.org/

HPO, https://hpo.jax.org/app/

ClinVar, https://www.ncbi.nlm.nih.gov/clinvar/

HGMD, http://www.hgmd.cf.ac.uk/ac/search.php

Mutalyzer, https://mutalyzer.nl/

### Supplementary information

Supplementary tables

Supplementary figure

## Data Availability

Additional data supporting the study are available from the corresponding author upon reasonable request.
