# Supplementary material for: Biallelic loss of function variants in FUZ result in an orofaciodigital syndrome
Source: Eur J Hum Genet. 2024 May 3;32(8):1022–6. doi: 10.1038/s41431-024-01619-6 (PMC11291644; doi:10.1038/s41431-024-01619-6)
Supplement: Supplementary file 1 — Supplementary tables [file 41431_2024_1619_MOESM1_ESM.docx]

**Supplementary tables**

**Biallelic loss of function variants in *FUZ* result in an orofaciodigital syndrome**

Swati Singh^1^, Sheela Nampoothiri^2^, Dhanya Lakshmi Narayanan^1^, Chakshu Chaudhry^3^, Sandesh Salvankar^3^, Katta M Girisha^1,3,4^

^1^Department of Medical Genetics, Kasturba Medical College, Manipal, Manipal Academy of Higher Education, Manipal, Karnataka, India

^2^Department of Paediatric Genetics, Amrita Institute of Medical Sciences and Research Centre, Kochi, India

^3^ Suma Genomics Private Limited, Manipal, India

^4^ Department of Genetics, College of Medicine and Health Sciences, Sultan Qaboos University, Muscat, Sultanate of Oman

**Correspondence:**

**Dr Katta M. Girisha**

Professor

Department of Genetics,

College of Medicine and Health Sciences,

Sultan Qaboos University Muscat, Sultanate of Oman and

Department of Medical Genetics,

Kasturba Medical College, Manipal,

Manipal Academy of Higher Education,

Manipal, Karnataka, India

Email address: [girish.katta@manipal.edu](mailto:girish.katta@manipal.edu)

**Supplementary table 1**: Regions of homozygosity in the proband 1. This variant c.601G>A (NC_000019.10:g.49809467C>T) resides in an autozygous region (19:49550972-50909765) of 1.36 Mb, indicating a shared ancestry.

| **#Chr** | **Begin** | **End** | **Size (Mb)** | **Nb_variants** | **Percentage_homozygosity** |
| --- | --- | --- | --- | --- | --- |
| chr1 | 55505668 | 57085063 | 1.58 | 34 | 97.06 |
| chr1 | 86529379 | 87633929 | 1.10 | 31 | 90.32 |
| chr2 | 70677819 | 75425623 | 4.75 | 117 | 99.15 |
| chr2 | 135745722 | 137640006 | 1.89 | 31 | 90.32 |
| chr2 | 186412410 | 187455278 | 1.04 | 34 | 88.24 |
| chr3 | 56658871 | 58553160 | 1.89 | 108 | 89.81 |
| chr3 | 78737962 | 88999950 | 10.26 | 51 | 90.20 |
| chr4 | 48170669 | 49237113 | 1.07 | 29 | 93.10 |
| chr4 | 118006307 | 119615544 | 1.61 | 32 | 93.75 |
| chr4 | 127544514 | 130824507 | 3.28 | 41 | 95.12 |
| chr5 | 114515588 | 115628120 | 1.11 | 34 | 91.18 |
| chr6 | 34078463 | 35705892 | 1.63 | 51 | 92.16 |
| chr6 | 99936251 | 101312104 | 1.38 | 26 | 88.46 |
| chr6 | 169730993 | 170949288 | 1.22 | 36 | 100.00 |
| chr9 | 127781259 | 130690412 | 2.91 | 79 | 91.14 |
| chr11 | 12231179 | 13514053 | 1.28 | 47 | 91.49 |
| chr11 | 57344024 | 58715285 | 1.37 | 41 | 92.68 |
| chr11 | 122944293 | 124253100 | 1.31 | 66 | 100.00 |
| chr11 | 124253170 | 133680714 | 9.43 | 183 | 96.17 |
| chr14 | 24529820 | 26900817 | 2.37 | 80 | 93.75 |
| chr14 | 61451589 | 64676098 | 3.22 | 44 | 88.64 |
| chr15 | 66257236 | 68116400 | 1.86 | 40 | 92.50 |
| chr19 | 48202125 | 49547378 | 1.35 | 182 | 97.80 |
| chr19 | 49550972 | 50909765 | 1.36 | 207 | 99.52 |
| chr20 | 23961350 | 25755672 | 1.79 | 42 | 100.00 |
| chr20 | 29637938 | 31673846 | 2.04 | 56 | 94.64 |

Variant (NC_000019.9:g.50312724C>T) in *FUZ* lies in highlighted (yellow) region. chr: chromosome

**Supplementary table 2:** Haplotype analysis revealed a shared haplotype between proband 1 and proband 2. This observation suggests a common lineage and the possibility of the variant being more prevalent in the Indian population.

| **Region of homozygosity** | | | **Proband 1** | **Proband 2** | |
| --- | --- | --- | --- | --- | --- |
| Chromosome | Position | Allele (Ref) | Allele (Hom) | Allele 1 | Allele 2 |
| chr19 | 50265549 | - | - | G | T |
| chr19 | 50265682 | - | - | C | CTT |
| chr19 | 50301844 | - | - | G | T |
| chr19 | 50304336 | A | G | G | A |
| chr19 | 50304392 | T | C | C | T |
| chr19 | 50304439 | T | C | C | T |
| chr19 | 50305688 | G | C | C | G |
| chr19 | 50308893 | T | C | C | T |
| chr19 | 50309615 | CCCT | C | C | CCCT |
| chr19   \|  \| \| --- \| | 50309860 | TC | T | T | TC |
| chr19   \|  \| \| --- \| | 50312688 | - | - | G | GCAGGGACCCCAC |
| chr19 | 50312724 | C | T | T | C |
| chr19 | 50314738 | A | C | C | A |
| chr19 | 50315423 | G | A | A | G |
| chr19 | 50315698 | A | C | C | A |
| chr19 | 50315773 | T | A | A | T |
| chr19 | 50316083 | C | T | T | C |
| chr19 | 50321510 | C | T | T | C |
| chr19 | 50321512 | - | - | G | C |
| chr19 | 50321981 | - | - | C | G |
| chr19 | 50322100 | - | - | G | A |
| chr19 | 50322111 | - | - | C | T |

Both the probands share a common haplotype within the genomic position range 50304336-50321510 that harbours the NC_000019.9:g.50312724C>T variant. Hom: homozygous; Het: heterozygous; red border indicates variants identified in our study.
