# Supplementary material for: Biallelic loss of function variants in FUZ result in an orofaciodigital syndrome
Source: Eur J Hum Genet. 2024 May 3;32(8):1022–6. doi: 10.1038/s41431-024-01619-6 (PMC11291644; doi:10.1038/s41431-024-01619-6)
Supplement: Supplementary file 2 — Supplementary figure [file 41431_2024_1619_MOESM2_ESM.pdf]

```
                                  p.(Glu201Lys)
Homo sapiens            WW-RLGTPEAVLLPWLVGSLPPQTAR     218
Pan troglodytes         WW-RLGTPEAVLLPWLVGSLPPQTAR     218
Pongo abelii            WW-RLGTPEAVLLPWLVGSLPPQTAR     218
Felis catus             WW-RLGTPEAVLLPWLVGSLPPQAAR     218
Rattus norvegicus       WW-RLGMPEAVLLPWLVGSLPPQAAR     218
Xenopus tropicalis      WW-RLSSQEVMLLCWLVASLAPHSSR     210
Drosophila melanogaster WW-DLDTRDRELLLFLLNSS-STMQH     219
Carassius auratus       WWSRLSSQEVVVLSVLVHSLSGASSC     217
                        **: **  *.  :  :*  *: *
```

Supplementary figure 1: Multiple sequence alignment of p.(Glu201Lys) in *FUZ* indicates that glutamine residue at 201 position is conserved among different vertebrates. An asterisk (*) represents fully conserved residue, colon (:) represents residues with strongly similar properties, and period (.) represents residues with weakly similar properties.

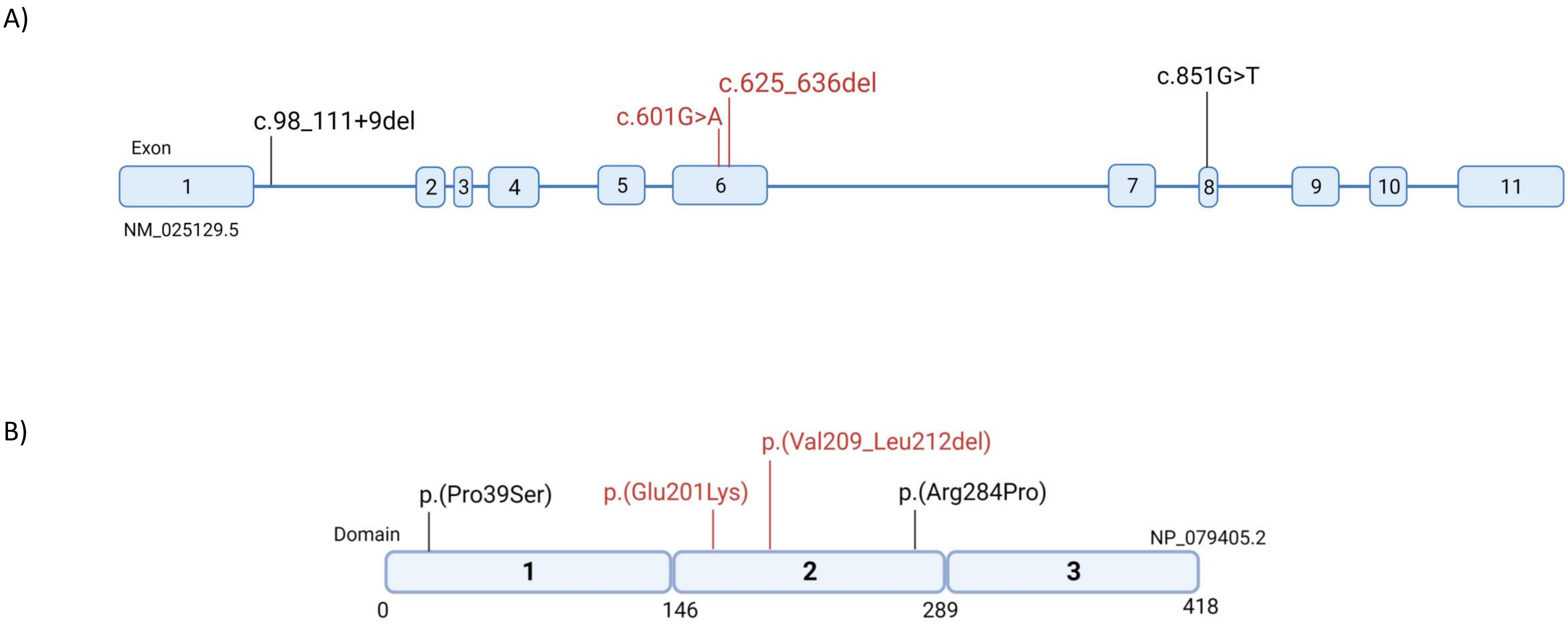

Supplementary figure 2: A) Cartoon illustration of structure of the gene (*FUZ*) and (B) domains of FUZ protein with disease-causing variants identified in individuals with FUZ related disorders. Novel biallelic variants identified in this study are coded in red.
